# Polarity and intracellular compartmentalization of *Drosophila *neurons

**DOI:** 10.1186/1749-8104-2-7

**Published:** 2007-04-30

**Authors:** Melissa M Rolls, Daisuke Satoh, Peter J Clyne, Astra L Henner, Tadashi Uemura, Chris Q Doe

**Affiliations:** 1 Institutes of Neuroscience and Molecular Biology, Howard Hughes Medical Institute, University of Oregon, Eugene, USA; 2 Department of Biochemistry and Molecular Biology, Penn State, University Park, USA; 3 Graduate School of Science, Kyoto University, Kyoto, Japan; 4 Department of Biochemistry and Biophysics, University of California, San Francisco, USA; 5 Graduate School of Biostudies, Kyoto University, Kyoto, Japan

## Abstract

**Background:**

Proper neuronal function depends on forming three primary subcellular compartments: axons, dendrites, and soma. Each compartment has a specialized function (the axon to send information, dendrites to receive information, and the soma is where most cellular components are produced). In mammalian neurons, each primary compartment has distinctive molecular and morphological features, as well as smaller domains, such as the axon initial segment, that have more specialized functions. How neuronal subcellular compartments are established and maintained is not well understood. Genetic studies in *Drosophila *have provided insight into other areas of neurobiology, but it is not known whether flies are a good system in which to study neuronal polarity as a comprehensive analysis of *Drosophila *neuronal subcellular organization has not been performed.

**Results:**

Here we use new and previously characterized markers to examine *Drosophila *neuronal compartments. We find that: axons and dendrites can accumulate different microtubule-binding proteins; protein synthesis machinery is concentrated in the cell body; pre- and post-synaptic sites localize to distinct regions of the neuron; and specializations similar to the initial segment are present. In addition, we track EB1-GFP dynamics and determine microtubules in axons and dendrites have opposite polarity.

**Conclusion:**

We conclude that *Drosophila *will be a powerful system to study the establishment and maintenance of neuronal compartments.

## Background

Since individual neurons were first observed, axons and dendrites have been recognized as distinct compartments. Dendrites were proposed to receive information, and axons to transmit it to other sites. Some general morphological features distinguish axons and dendrites. Dendrites are typically shorter than axons, taper as they leave the cell body, and decrease in size as they branch. The diameter of axons is relatively constant, and does not decrease with branching [1]. In the last several decades, molecular differences, including the presence of different membrane and cytoskeletal proteins in neuron subregions, have been added to these original morphological observations [1,2].

One type of molecular difference that is likely to be fundamental to neuronal polarity is the distinction between the axonal and dendritic microtubule cytoskeleton. Specific sets of microtubule-binding proteins are found in axons and dendrites. For example, the microtubule-binding protein MAP2 is enriched in dendrites, while dephospho-tau is enriched in axons. The microtubules themselves are also organized differently in axons and dendrites. In axons, microtubules are oriented with their plus-ends distal to the cell body, while in proximal dendrites microtubule polarity is mixed [3]. It is thought that differences in the microtubule cytoskeleton contribute to polarized trafficking to axons and dendrites.

Membrane proteins, including neurotransmitter receptors, ion channels, and adhesion proteins, can also be selectively targeted to axons or dendrites [1,4]. To help maintain distinct axonal and somatodendritic plasma membranes, a diffusion barrier is present in the initial segment of the axon [5]. The implications of selective membrane protein targeting to axons and dendrites for neuronal function are profound. For example, the presence of different receptors and adhesion molecules on axons and dendrites means they can be guided by different external signals as they grow.

Another major type of neuronal compartmentalization is localization of protein synthesis machinery. The bulk of ribosomes, RNA, and other proteins required for protein synthesis are localized to the cell body and proximal dendrites [1]. This type of specialization is easy to see by electron microscopy, but its significance is not well-understood. Major advances in recent years have centered on transport of specific RNAs, usually with associated ribosomes and other translation proteins, into dendrites [6]. In general, ribosomes and RNAs are rare in axons, although in specific circumstances, for example during axon pathfinding, axonal RNAs do have an important role [7]. Nothing is known about the mechanism that keeps most RNAs and other protein synthesis machinery out of axons and dendrites.

In addition to the major division of a neuron into axons, dendrites and soma, further regional specialization can exist. For example, concentration of voltage-gated sodium channels in the beginning of the axon permits this region to function as a decision point in action potential generation [8].

The types of neuronal compartmentalization described above were identified primarily by analysis of mammalian neurons. In particular, much of the work on regional molecular differences in neurons has been carried out in primary cultures of rodent neurons. While these culture systems are extremely useful for studying neuronal polarity, it would be beneficial to have an alternative system with different strengths, for example, the ability to use genetics and observe neurons *in vivo*.

Genetic analysis of neural development in both *Drosophila *and *Caenorhabditis elegans *has already made profound contributions to studying axon pathfinding [9], and could prove similarly useful for neuronal polarity. However, invertebrate neuronal organization has been viewed as fundamentally different from that of vertebrate neurons [1,10]. One of the most widely cited reviews on neuronal polarity suggested of invertebrate neurons that "the organization of their axonal and dendritic domains is sufficiently different from that in vertebrate neurons to suggest that the details of molecular sorting may also differ" [1]. In this study we assemble a set of new and previously characterized markers to analyze the molecular compartmentalization of two types of *Drosophila *neurons. Although some of the markers have been analyzed in isolation before, we believe that analyzing their localization together provides a much more complete view of neuronal organization in *Drosophila*. We also use live imaging to directly analyze axonal and dendritic microtubule orientation for the first time in an invertebrate.

**Figure 1:**
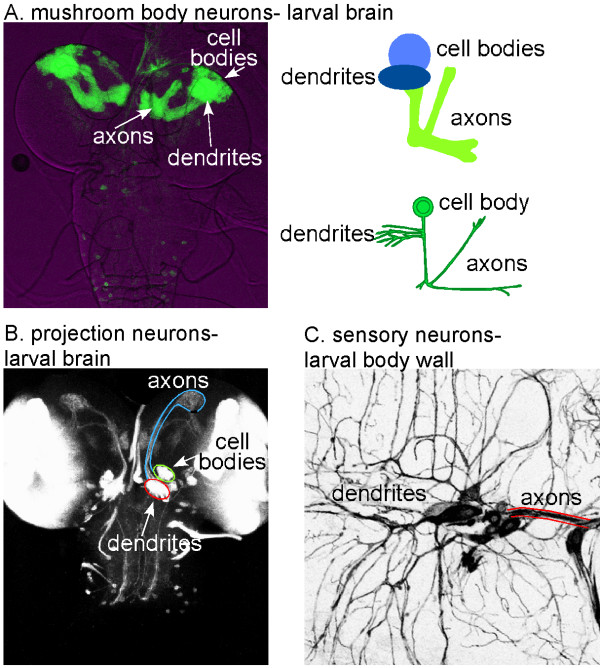
Overview of three types of *Drosophila *neurons. **(a) **Larval mushroom body interneurons. These neurons were marked by crossing flies containing the mushroom body Gal4 driver 201Y with flies containing a UAS-mCD8-GFP transgene. Brains were dissected from third instar larval progeny. A projection of a confocal image stack is shown. The structure is shown schematically to the right, and a diagram of a single neuron is shown at the lower right. **(b) **Olfactory projection interneurons. These neurons were marked by crossing GH146 Gal4 and UAS-mCD8-GFP flies. Brains from third instar progeny were dissected and a projection of a confocal stack is shown. The GH146 Gal4 driver also expresses in the optic lobe, which accounts for the bright GFP in the lateral region of the brain. Different compartments of the projection neurons are outlined. The axon projections are outlined to their synapses on the mushroom body calyx, but the continuation to the lateral horn is not outlined as the axons become more difficult to follow. **(c) **Sensory neurons in the dorsal cluster, including dendritic arborization neurons. Neurons were labeled by crossing elav-Gal4 and UAS-mCD8-GFP flies. Confocal microscopy was used to image just below the cuticle of whole, live early second instar progeny; a projection is shown with the scale inverted for clarity. Cell bodies are in the center of the image. Anterior is left and dorsal is up. Dendrites project dorsally and branch extensively just under the cuticle. Axons project down and form a tight bundle.

For analysis of neuronal compartmentalization, we studied two types of larval interneurons: Kenyon cells of the mushroom body, and projection neurons of the antennal lobe. The development and morphology of both of these types of neurons has been previously characterized [11,12], and both can be specifically labeled with the Gal4-UAS binary expression system [13] (Figure 1a, b). The mushroom body is required for olfactory learning during both larval and adult stages [14], and projection neurons transmit signals from the antennal lobe glomeruli to the mushroom body calyx (dendrites) and lateral horn, where the information is further processed [15]. Visualization of both types of neurons in the larval brain shows that they have axons and dendrites that occupy specific regions in the brain (Figure 1a, b). We obtained similar results in both types of neurons, indicating that they are likely to be broadly applicable to *Drosophila *neurons. For live imaging, we used the larval peripheral nervous system, as it lies close to the surface and can be easily visualized in whole animals (Figure 1c).

We identified a set of markers that localize to different compartments in *Drosophila *neurons, and demonstrate that the major types of subcellular compartmentalization present in mammalian neurons can also be found in *Drosophila *neurons. In addition, we found that microtubules are oriented differently in axons and dendrites. We conclude that *Drosophila *will be a powerful system in which to study neuronal compartmentalization.

## Results

### Axon and dendrite microtubules have different properties and orientation

**Figure 2:**
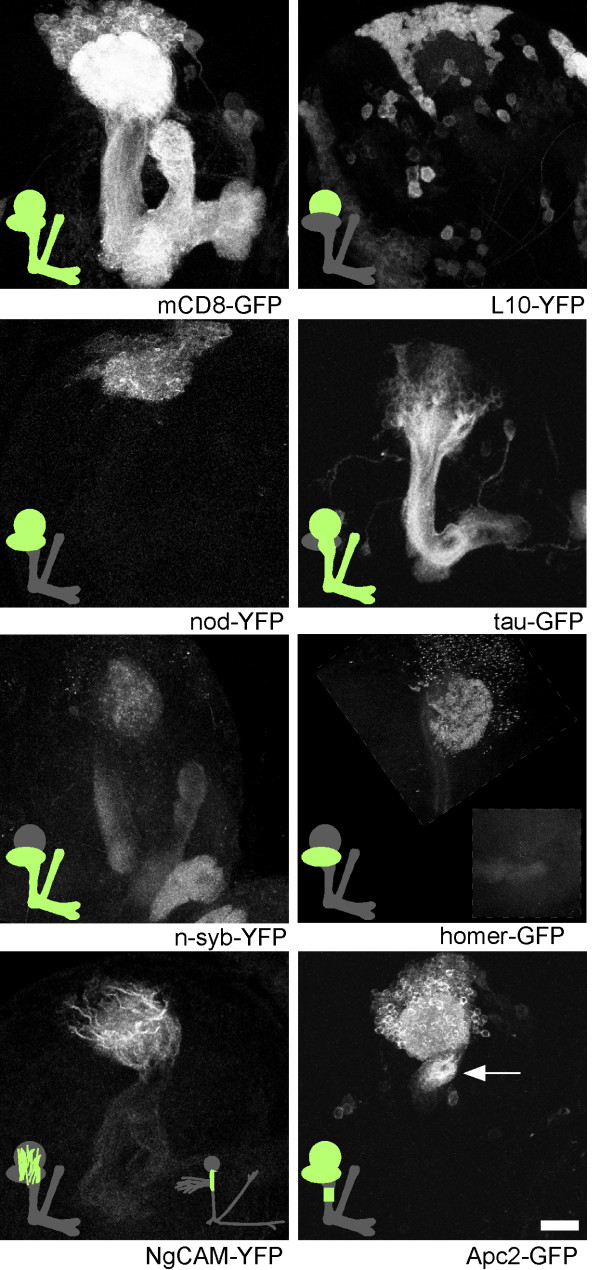
Mushroom body neurons are divided into molecularly distinct compartments. Mushroom body neurons expressing tagged markers were generated by crossing flies containing mushroom body Gal4 drivers with flies carrying UAS-controlled markers. The 201Y Gal4 was used in all panels except L10-YFP and homer-GFP, for which the OK107 Gal4 was used. 201Y drives expression in a large subset of mushroom body neurons [54, 55], and OK107 drives expression in all mushroom body neurons [11] as well as the optic lobe. Brains were dissected from third instar larvae, fixed and analyzed by confocal microscopy. All panels represent confocal projections through the entire mushroom body, except homer-GFP, which shows the cell bodies, dendrites and proximal axons, with distal axons in the inset. Low expression levels of n-syb-YFP expression were generated by using a 2XUAS vector, and low levels of homer-GFP were generated by raising larvae at 18°C. Arrow points to region of proximal axon in which Apc-2 GFP was localized. Scale bar at lower right is 20 μm.

**Figure 3:**
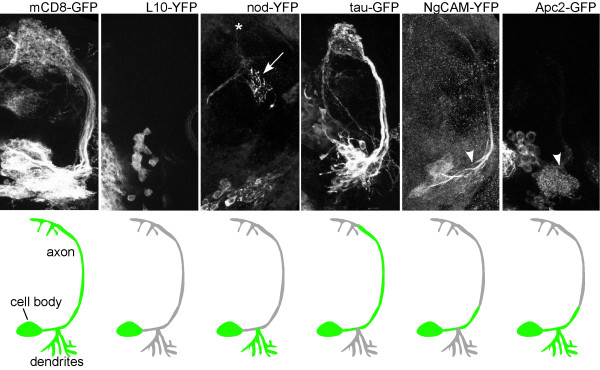
Olfactory projection neurons have molecularly distinct compartments. Tagged markers were expressed in olfactory projection neurons using the GH146 Gal4 driver, which is specific to these neurons and a few other scattered groups of neurons in the brain [56]. Brains were dissected from third instar larvae and fixed. Signal in nod-YFP and NgCAM-YFP panels was amplified by staining with GFP antibodies. Confocal projections through the region occupied by projection neurons are shown. The arrow in the nod-YFP panel represents fluorescence from other neurons; the asterisk indicates the projection neuron axons terminate; arrowheads indicate the beginning of the axons, just distal to the dendrite branch point. Scale bar is 20 μm.

To determine whether microtubule-binding proteins can be preferentially localized to axons and dendrites in flies, we examined exogenous and endogenous microtubule-binding proteins in the *Drosophila *larval brain. The two exogenous proteins we examined were: tau-green fluorescent protein (tau-GFP) and nod-yellow fluorescent protein (nod-YFP). Some reports have suggested that tagged versions of the microtubule binding domain of bovine tau preferentially label axons in flies [16], although others have also reported dendrite localization [17]. We examined the distribution of one of these tagged bovine tau proteins, tau-myc-GFP (which we call tau-GFP for simplicity) [17] in mushroom body and projection neurons (Figures 2 and 3). In both mushroom body and projection neurons, tau-GFP was abundant in the main axon tracts. It was less abundant in distal axons and dendrites. For comparison, mCD8-GFP (Figures 2 and 3) is present in all neuronal compartments. Thus, tau-GFP preferentially labels proximal axons. Fusion proteins that consist of the nod motor domain, kinesin coiled-coil, and a tag have previously been localized to dendrites [16,18-21]. To confirm that nod fusions label dendrites specifically, we expressed nod-YFP in mushroom body and projection neurons. In both cases nod-YFP localized clearly to dendrites but not axons (Figures 2 and 3). Thus, two exogenous microtubule-binding proteins, tau-GFP and nod-YFP, localized to different neuronal compartments, indicating that axonal and dendritic microtubules have distinct features in *Drosophila*.

**Figure 4:**
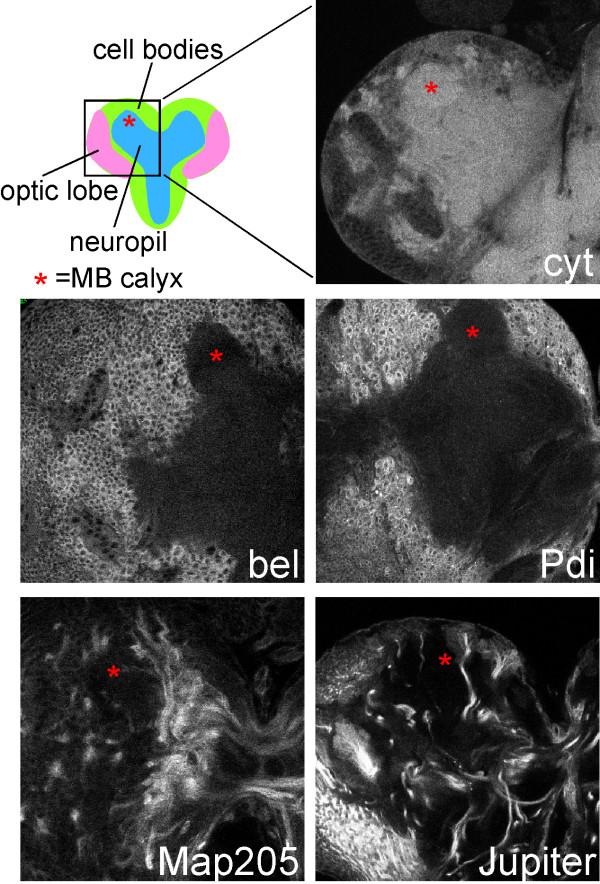
Tagged endogenous proteins localize to distinct neuronal compartments. Homozygous fly lines containing GFP transposon insertions were imaged by confocal microscopy. Single confocal sections of third instar brains are shown. The images were all acquired at the depth of the mushroom body calyx; the calyx region is indicated in each image with an asterisk. A diagram of a section through a third instar brain is shown at the upper left. One brain lobe (boxed region) is shown in each panel. At the top right an example of a GFP transposon insertion that yields cytoplasmic fluorescence in the cell body, axons and dendrites is shown. The *bel*, *Pdi*, and *Map205 *genes all contain Wee-P [22] insertions, and the Jupiter line was previously described [23]. Scale bar at lower left is 50 μm.

We also examined the localization of two *Drosophila *microtubule-binding proteins under control of their own promoters. We performed a protein trap screen as described [22] to identify proteins targeted to specific regions of neurons. Fly lines isolated from this screen contain transposons with the GFP coding sequence inserted into the genome. Insertions within genes result in the GFP coding sequence being spliced into the mRNA, and generate tagged proteins from the genomic locus. GFP-Map205 was identified as a line with strong axonal GFP. Another microtubule binding protein, GFP-Jupiter, was previously isolated [23]. Both protein trap lines exhibited strong GFP fluorescence in a subset of neurons. Like tau-GFP, both *Drosophila *microtubule binding proteins were highly concentrated in axons (Figure 4), although the relative amounts of tagged protein in axons and dendrites were harder to compare when expression was scattered throughout the brain. This preliminary localization data suggest that both are good candidates for endogenous axon-specific microtubule binding proteins.

### Axon and dendrite microtubules have opposite orientation

As a difference in microtubule orientation in axons and dendrites is a fundamental aspect of vertebrate neuronal polarity, we wished to determine exactly how microtubules are arranged in fly neurons. The dendritic localization of nod fusion proteins, which are believed to act as minus-end directed motors, has been used to argue that *Drosophila *dendrites are likely to have minus-ends distal to the cell body like vertebrate dendrites [18]. However, direct analysis of the orientation of individual microtubules has not been performed in any invertebrate neuron.

Analysis of a microtubule plus-end tracking protein was previously used in mammalian neurons to confirm that axon and dendrite microtubules have different orientations [24]. As these proteins generally bind only to the growing plus ends of microtubules, microtubule orientation can be inferred from the direction of movement of a tagged plus-end binding protein. The peripheral nervous system of the *Drosophila *larva is well-suited to live imaging and has been previously used to study actin dynamics [20]. We therefore expressed the plus-end binding protein EB1-GFP throughout the nervous system using an elav-Gal4 driver, and performed time lapse imaging of the dorsal cluster of the peripheral nervous system (Figure 1c) in live, whole, early L2 larvae. We primarily analyzed EB1-GFP dynamics in axons and dendrites of dendritic arborization neurons, which have highly branched dendrites [25-27].

**Figure 5:**
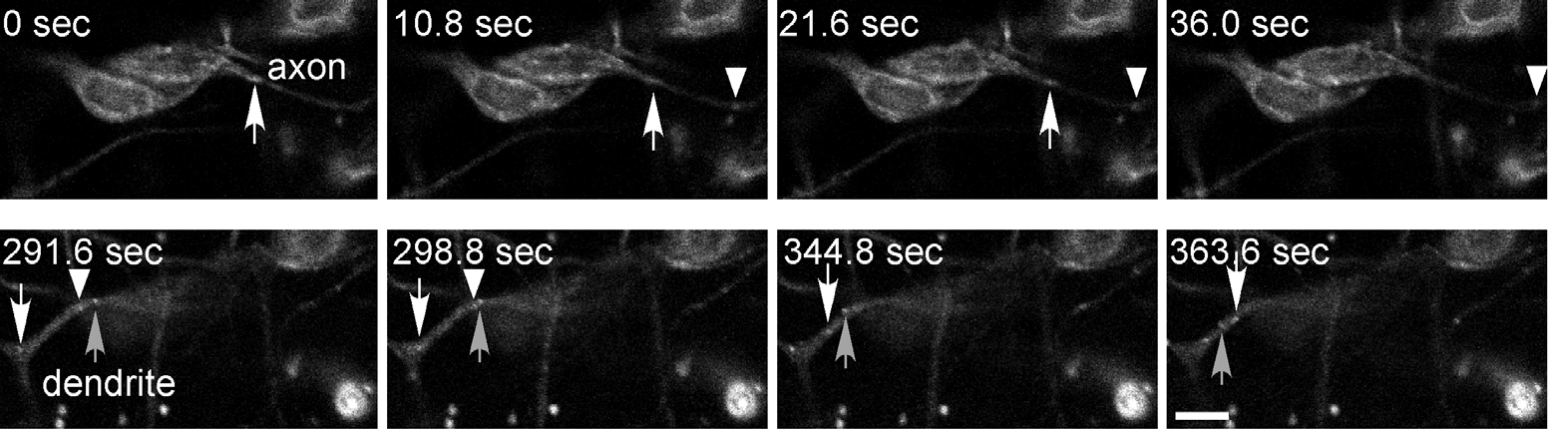
Determination of microtubule polarity in axons and dendrites using EB1-GFP dynamics. Flies containing an elav-Gal4 transgene were crossed to flies with a UAS-EB1-GFP transgene. Progeny were aged to early L2 and whole larvae were mounted for imaging. Single confocal images were acquired every 3.6 seconds. Additional data file 1 is a movie that shows all images acquired. Frames from two portions of the movie were selected for still images. The top row shows EB1-GFP fluorescence in an axon of a dendritic arborization neuron. The arrow and arrowhead track two different EB1-GFP dots as they move away from the cell body. The bottom row shows the dendrite from the same neuron as the top panels; these images were acquired at a different plane of focus. Three different EB1-GFP dots are tracked. The one indicated with the upward-pointing arrow moves away from the cell body and the other two move towards the cell body. The figure is shown with anterior up and dorsal to the left. Scale bar is 5 μm.

EB1-GFP dots were clearly seen moving in the cell body, axons, and dendrites (Figure 5 and Additional data file 1). Movements of EB1-GFP dots were consistent with the tagged protein only binding to growing microtubule plus ends: an individual dot that could be followed through multiple frames never changed direction, and after a dot tracked through a particular region of an axon or dendrite and disappeared, often a dot with a similar track appeared several frames later. All EB1-GFP dots in axons moved away from the cell body (24 out of 24; only dots that could be followed in three consecutive frames were counted; Table 1). An example of an axon with two different EB1-GFP dots is shown in the upper panels of Figure 5. In dendrites the movements were very different. The vast majority of dots moved toward the cell body (53 out of 55; dots present in 3+ frames). Occasionally, dendrites were observed that had dots moving in opposite directions (Figure 5, lower panels, and Additional data file 1).

**Table 1:** Summary of EB1-GFP dynamics in dendritic arborization neurons

	Movements in axons	Movements in dendrites
Away from cell body	24	2
Towards cell body	0	53

Movies of EB1-GFP movements in early L2 larvae were collected as described in Figure 5. For quantification, dots that could be tracked for three or more frames were counted, and their direction of movement in axons or dendrites was recorded.

Thus, in fly dendritic arborization neurons, axonal microtubules were oriented with plus ends distal to the cell body. Most dendritic microtubules were oriented with minus ends distal to the cell body, although dendrite microtubules were sometimes mixed in orientation.

### Protein synthesis machinery is concentrated in the cell body

In mammalian neurons, the bulk of protein synthesis takes place in the cell body. We generated a tagged ribosomal protein, L10-YFP to determine where protein synthesis takes place in *Drosophila *neurons. When expressed in both mushroom body (Figure 2) and projection (Figure 3) neurons, L10-YFP was concentrated in the cell body, with only very faint signal present in neuropil. Within the cell body it was seen in the cytoplasm, and in some cells it was also present in the nucleus, where ribosomes are assembled. Faint signal in dendrites may represent ribosomes or free L10-YFP.

To confirm that the L10-YFP marker represents the localization of endogenous protein synthesis machinery, we compared its localization to two proteins identified in our protein trap screen. One of our GFP insertions was in the *bel *gene. Bel is a DEAD-box protein that is likely to function as an RNA-binding protein with a role in translation initiation [28]. The GFP transposon insertion we isolated was homozygous viable. As *bel *is an essential gene [28], this means that the GFP-tagged protein that is generated from the insertion is likely to be functional, and thus the localization of the protein trap very likely represents that of the endogenous protein. Bel was broadly expressed, and in neurons it localized to the cell body (Figure 4). A protein trap line in which GFP localized to the cytoplasm in all neuronal compartments is shown for comparison in the upper right panel of Figure 4. The other insertion we analyzed was in the *pdi *gene. Pdi is a chaperone that resides in the endoplasmic reticulum (ER) lumen and is involved in processing newly synthesized membrane and secretory proteins. GFP-Pdi was also seen throughout the brain and was highly concentrated in the neuron cell body (Figure 4). Within the cell body, GFP-Pdi was brightest in the perinuclear region, which is consistent with localization to the ER. Thus, an exogenous protein synthesis protein, L10-YFP, and two endogenous ones, GFP-Bel and GFP-Pdi, were all concentrated in the neuronal cell body, suggesting that the bulk of protein synthesis takes place there.

### Pre- and post-synaptic sites are localized to different regions of mushroom body neurons

One of the longest recognized forms of neuronal compartmentalization is concentration of postsynaptic sites to dendrites and presynaptic sites to axons. To determine whether excitatory synaptic inputs are received in dendrites, we analyzed the distribution of the postsynaptic marker homer-GFP in mushroom body neurons. Mammalian homer proteins bind metabotropic glutamate receptors and Shank, which forms a complex with NMDA glutamate receptors. The *Drosophila *homer protein also binds Shank and localizes to postsynaptic sites [29]. Homer-GFP localizes similarly to endogenous homer [29]. In brains that expressed homer-GFP at low levels in the mushroom body (see Materials and methods), fluorescence was confined to dendrites and dots in the cell body (likely to be the Golgi based on previous analysis [29]), and was not present in axons (Figure 2). The pattern of homer-GFP fluorescence in the mushroom body dendrite region (calyx) was similar to the strongest regions of staining with anti-Dlg and anti-Scrib immunofluorescence (not shown); both of these proteins are concentrated at postsynaptic sites. Thus, a marker of excitatory postsynaptic sites was polarized to dendrites.

**Figure 6:**
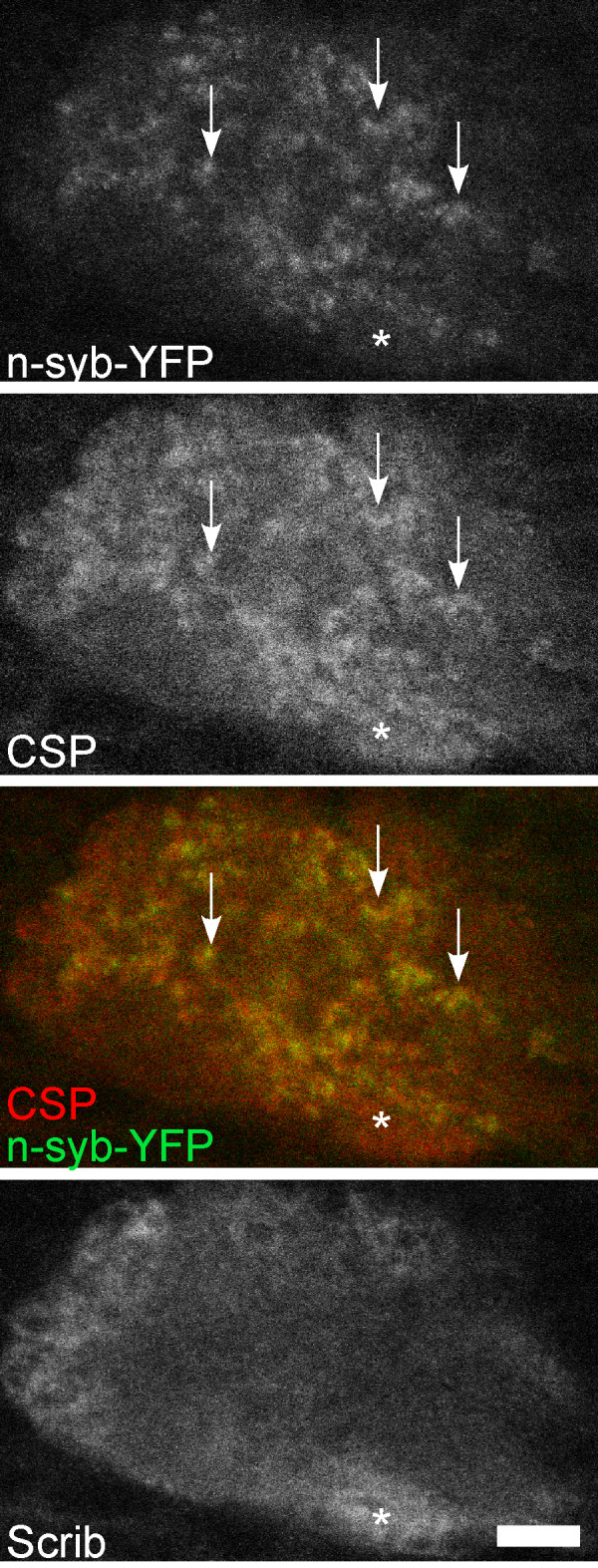
The n-syb-YFP marker is localized to synaptic vesicles in mushroom body dendrites. Brains from third instar larvae expressing n-syb-YFP in mushroom body neurons were dissected and immunostained for CSP and Scrib. A single confocal section through the mushroom body calyx is shown. The arrows show clusters of n-syb-YFP dots that colocalize with CSP. The asterisk shows a region that has Scrib and CSP staining, but no n-syb-YFP. Scale bar is 5 μm.

We generated low expression level n-synaptobrevin-YFP (n-syb-YFP) transgenic flies to specifically label synaptic vesicles in the mushroom body. These transgenes had either one or two UAS sites upstream of the transcriptional start site, rather than the usual five. Spots of fluorescence were seen in the axons and dendrites; very little was present in the cell body (Figure 2). To confirm that the dots of fluorescence represented synaptic vesicles, we stained brains expressing n-syb-YFP in the mushroom body with cysteine string protein (CSP) and Scrib antibodies. CSP is a synaptic vesicle protein that is abundant in all presynaptic terminals [30], and Scrib is a synaptic protein that is concentrated in the postsynaptic terminal [31]. Many of the n-syb-YFP dots in the calyx region of the brain, which contains mushroom body dendrites, overlapped with the CSP, but not the Scrib, pattern (arrows in Figure 6), indicating that n-syb-YFP is present in synaptic vesicles. Note that some regions of the calyx that are positive for CSP do not contain n-syb-YFP (asterisk in Figure 6). Extrinsic neurons, such as olfactory projection neurons, synapse onto mushroom body dendrites in the calyx, and so it is expected that not all synaptic vesicles would be accounted for by n-syb-YFP expressed in mushroom body neurons. Having verified that our low expression n-syb-YFP marker colocalized with synaptic vesicles, we concluded that synaptic vesicles are present in axons and dendrites of mushroom body neurons. We were unable to generate appropriate levels of expression of pre- or post-synaptic markers for analysis in projection neurons.

### Regional cytoskeletal and membrane specialization in the proximal axon

Thus far, we have concentrated on basic differences between axons, dendrites and the cell body. One of the most important further regional specializations is the axon initial segment, which contains specific arrangements of membrane and cytoskeletal proteins. In our survey of marker localization in *Drosophila *neurons, we identified two proteins that showed very distinctive localization to the proximal neurite and axon.

NgCAM-YFP expressed at low levels (see Materials and methods) was concentrated at the beginning of the neurite in mushroom body neurons (Figure 2). In projection neurons, NgCAM-YFP was clearly seen in the primary neurite before the dendrites branched off, and in the proximal axon beyond the dendrite branch point (Figure 3; arrowhead indicates start of axon, beyond dendrite branch point). Much fainter fluorescence was present in distal axons and dendrites. NgCAM is a chick neural cell adhesion molecule that is selectively localized to axons when expressed in cultured hippocampal neurons [32], and can be tethered by ankyrins in the initial segment [33].

Another tagged protein, Apc2-GFP, was targeted to the proximal region of *Drosophila *axons. In both mushroom body and projection neurons, Apc2-GFP was present in the cell body and dendrites (Figures 2 and 3). In mushroom body neurons it localized to just one region of the axons, near the beginning of the peduncle (arrow in Figure 2). In projection neurons, Apc2-GFP also localized to the proximal axon (Figure 3), but the pattern was not quite as striking as in the mushroom body, probably because Apc2-GFP expression levels were lower in the projection neurons. The region of the proximal axon to which Apc2-GFP was localized in mushroom body neurons was just distal to the stretch of proximal neurites in which NgCAM-YFP was concentrated. Adenomatous polyposis coli (APC) proteins regulate wingless signaling, and they also bind a number of cytoskeletal proteins, including microtubules and the plus-end microtubule binding protein EB1 [34]. The localization of two cytoskeleton-interacting proteins to the proximal axon in flies indicates that the *Drosophila *axon is divided into domains with specialized cytoskeletal properties. It will be interesting to determine whether this region is functionally similar to the vertebrate axon initial segment.

## Discussion

Many aspects of invertebrate neuronal organization have not been previously examined, and so some have considered it to be different than that of mammals [1,10], while others have concluded it is similar [16]. To determine how *Drosophila *neurons are organized, we have used several approaches, including analysis of a set of tagged markers expressed in *Drosophila *central neurons.

We chose analysis of tagged markers as our primary assay because we could express them in single types of neurons and analyze their distribution unambiguously. Interpretation of antibody staining in complex central synaptic regions can be very difficult. For example, in the mushroom body calyx resolving pre- and postsynaptic localization often requires immuno-electron microscopy. We also used analysis of tagged endogenous proteins and live imaging to obtain additional information about localization of protein synthesis and microtubule organization. We found fly neurons exhibit the major kinds of compartmentalization present in mammalian neurons. *Drosophila *should, therefore, be an extremely powerful system in which to study neuronal subcellular organization.

### Microtubule cytoskeleton

A fundamental aspect of mammalian neuronal polarity is distinct axonal and dendritic microtubule organization. Axon and dendrite microtubules differ in two major ways: microtubule binding proteins and filament orientation. We found evidence for both types of difference in *Drosophila*. We confirmed that two exogenous microtubule binding proteins, nod-YFP and tau-GFP, have different preferences for axons and dendrites. In addition, we found two endogenous *Drosophila *microtubule binding proteins, Jupiter and Map205, that are good candidates for axon-specific microtubule binding proteins. We also examined microtubule orientation in living *Drosophila *axons and dendrites using plus-end microtubule binding protein dynamics.

In mammalian cultured neurons and frog mitral cells, electron microscope analysis of hook labeled microtubules was initially used to analyze microtubule orientation [3,35]. Subsequently, analysis of a microtubule plus-end tracking protein, EB3-GFP, was used to determine microtubule orientation in cultured Purkinje and hippocampal neurons [24]. Both methods yielded similar results: in axons most plus ends were oriented away from the cell body; while in proximal dendrites microtubules had mixed orientation with slightly more plus ends distal to the cell body [3,24,35]. The concurrence of EB3-GFP dynamics and hook labeling may mean that most microtubules in axons and dendrites are dynamic, as EB3-GFP labels only growing plus ends, while all microtubules should be accessible to hook formation. It also means that we have every reason to believe that EB1-GFP dynamics in *Drosophila *axons and dendrites is a valid representation of microtubule orientation.

We found that microtubules in axons were arranged with their plus ends distal to the cell body, as in vertebrates. In the long, branched dendrites of dendritic arborization sensory neurons, most microtubules were arranged with their minus ends distal to the cell body. In occasional dendrites, mixed orientation microtubules were observed. This difference in microtubule orientation between axons and dendrites is consistent with the selective localization of nod motor domain fusion proteins to dendrites. The predominance of minus-end out microtubules in dendrites is surprising. Current models of traffic into dendrites rely on plus-end directed motors [36,37], but we predict that minus-end directed motors will be important for anterograde traffic in, at least, *Drosophila *dendritic arborization dendrites. It will be interesting to determine whether all *Drosophila *dendrites share this microtubule organization, or if it is specific to the peripheral nervous system.

### Protein synthesis

In *Drosophila *neurons we found protein synthesis machinery, including ribosomes, was highly concentrated in the neuron cell body. Others have reported the presence of RNA granules in *Drosophila *dendrites [38], so it is likely that some specific RNAs are transported into dendrites as in mammals. Concentration of the bulk of ribosomes to the neuron cell body has also been observed in *C. elegans *neurons [39]. In mammalian neurons, protein synthesis machinery is also concentrated in the cell body, but can extend into the proximal dendrite [1]. Thus, in all mature neurons so far examined, very little protein synthesis machinery is present in axons or distal dendrites. The fluorescence images of L10-YFP in Figures 2 and 3 show that, although this form of neuronal compartmentalization has been studied very little, it is extremely striking. The strong concentration difference of ribosomes between the cell body and neuropil indicates that there may be an active mechanism that restricts the bulk of protein synthesis machinery to the cell body.

One of the endogenous proteins that we found highly concentrated in the cell body was Pdi. Pdi is localized to the lumen of the ER. In all neurons previously examined, including Purkinje cells and *C. elegans *neurons, the ER extends from the cell body throughout axons and dendrites, and is believed to be continuous [39,40]. We therefore also predict that a mechanism exists to concentrate proteins inside the ER to the neuron cell body.

### Synaptic components

In mammalian motor and hippocampal neurons, presynaptic components are restricted to axons and postsynaptic components to dendrites, but in some mammalian interneurons, dendrites can contain synaptic vesicles and be presynaptic [10]. *Drosophila *embryonic motor neurons are similar to mammalian motor neurons: a neurotransmitter receptor is exclusively localized to dendrites, and a synaptic vesicle marker is restricted to axons [16]. We found that a postsynaptic marker was exclusively targeted to dendrites in mushroom body neurons, while a synaptic vesicle marker was present in dendrites and axons. Both mammals and flies, therefore, have different classes of neurons with distinct synaptic arrangements. Little is known about how non-classic synapses, including dendro-dendritic, are assembled. *Drosophila *has already proven useful for studying 'traditional' synapses, like the neuromuscular junction [41], and could be useful for understanding other synaptic arrangements. One interesting question is: how are synaptic vesicles targeted to dendrites in mushroom bodies, but excluded from dendrites of motor neurons?

### Plasma membrane proteins

In mammalian neurons, different non-synaptic plasma membrane proteins are localized to axons and dendrites. Examples include cell adhesion proteins and ion channels [1]. In *Drosophila*, non-synaptic plasma membrane proteins completely specific to axons or dendrites have not yet been identified. In part, this is because these experiments are easier to interpret using cultured neurons in which the plasma membranes are not contacting other cells, and in flies a fully polarized neuronal culture system has not been described. We localized one exogenous transmembrane adhesion protein, NgCAM-YFP, to the primary neurite and proximal axon, but did not identify more broadly localized axonal or dendritic proteins. These are, however, likely to exist in flies. One good candidate is the sodium channel pickpocket, which is present in a subset of dendritic arborization neuron dendrites [42].

### Do *Drosophila *neurons have an initial segment?

The mammalian axon initial segment is a distinctive region of the axon that is important in generation of action potentials and protein sorting within the neuron. At the ultrastructural level, it can be recognized by an electron-dense coating under the plasma membrane and bundled microtubules [10]. At the molecular level, the initial segment contains a specialized spectrin and ankyrin G submembrane skeleton that is important for localizing channels and other plasma membrane proteins. This same network forms the basis of a diffusion barrier that isolates the plasma membrane in the axon from that in the soma [5]. The microtubule cytoskeleton in the initial segment has also been suggested to be important for selective sorting to axons and dendrites [43].

While concentration of cytoskeletal (see tau-GFP in Figures 2 and 3) and membrane proteins [44] to unbranched or fasciculated subregions of axons has been observed in flies, it has not been clear whether flies have an initial segment similar to that in mammals. Many *Drosophila *neurons also generate action potentials, but the precise region in which they are initiated has not been determined [45]. *Drosophila *do possess a neuronal ankyrin that has axonal and somatic isoforms, but the axonal isoform has not been noted to be restricted to a particular domain [46,47]. Nor have specific membrane proteins been previously identified in the proximal axon, and the motif in ion channels that binds to ankyrin in the vertebrate axon initial segment is absent in insects [48].

Our data suggest that *Drosophila *neurons may have a domain similar to the mammalian axon initial segment. NgCAM-YFP localized to the proximal neurite and axon in mushroom body and projection neurons. In mammalian neurons, NgCAM seems to have two mechanisms of localization: an ankyrin-independent one that localizes it along the length of the axon, and an ankyrin-dependent one that anchors it at the initial segment [33]. One possible explanation for the localization we see in flies is that the ankyrin-independent targeting signal is not recognized, but NgCAM can be concentrated in the proximal axon by binding to a special submembrane skeleton analogous to the ankyrin G domain in the initial segment. We also observed localization of Apc2-GFP to the proximal axon. APC family members bind cytoskeletal proteins, including EB1. Overexpressed EB1-YFP localizes to the initial segment in hippocampal neurons, and may recognize a local microtubule specialization important for directional transport within neurons [43]. The localization of overexpressed Apc2-GFP we observe may reflect a similar microtubule organization in fly proximal axons. We therefore suggest that flies may have actin and microtubule cytoskeletal specializations in the proximal neurite and axon that are similar to those in the mammalian axon initial segment.

## Conclusion

We have found evidence for many types of regional neuronal specialization in *Drosophila*, and conclude that *Drosophila *and mammalian neurons are compartmentalized in the same ways. We have also generated a number of useful tools for following protein targeting in fly neurons, which, in conjunction with the sophisticated genetic techniques that can be used to manipulate *Drosophila*, mean that this should be a powerful system for studying neuronal polarity. Questions that could be addressed include: how is protein synthesis machinery concentrated in the cell body? How are axon domains established? What regulates the polarity of microtubules in axons and dendrites?

## Materials and methods

### Fly stocks

Bloomington Stock Center provided the following stocks: OK107-Gal4, 201Y-Gal4, elav-Gal4, and UAS:Apc2-GFP. GH146-Gal4 was kindly provided by Reinhard Stocker, UAS:tau-myc-GFP by Shingo Yoshikawa, and UAS:homer-myc-GFP by Ulrich Thomas. P{PTT-GA}Jupiter [G147] was provided by Flytrap [49]. UAS:nod-YFP, 2XUAS:n-syb-YFP, UAS:NgCAM-YFP, UAS:EB1-GFP, P{Wee}bel [0–139], P{Wee}Pdi [1–149], and P{Wee}Map205 [382] were generated for this study.

### P element construction

For tagging proteins with three copies of YFP, a modified pUAST [13] vector was designed. In 3XYFP-CT a polylinker was placed before three copies of the YFP coding sequence. UAS:nod-YFP was generated by PCR amplifying the nod motor domain and kinesin coiled coil sequence from pnod:lacZ [18], which was generously provided by Ira Clark; this PCR product was cloned into 3XYFP-CT. Tagged NgCAM was generated by PCR amplifying the NgCAM coding sequence from JPA7-NgCAMYFP, kindly provided by Gary Banker, and cloning it into 3XYFP-CT. A suboptimal ribosome binding site (TGAAAG) [50] was included in the upstream primer to reduce levels of translated protein. 2XUAS:n-syb-YFP was generated using a modified version of 3XYFP-CT in which the five UAS sequences were removed and replaced with two copies of the UAS sequence. Full length n-syb was PCR amplified from an expressed sequence tag clone (GH04664), and cloned into the 2XUAS-3XYFP-CT vector. The UAS:EB1-GFP P element was created by inserting cDNA of enhanced GFP fused to the carboxy-terminal end of *Drosophila *EB1 [51] into pUAST.

### Immunofluorescent staining of larval brains

Third instar larval brains were dissected in Schneider's medium and fixed for 20 minutes with 4% paraformaldehyde in phosphate-buffered saline (PBS). They were then washed several times with block buffer (PBS/1% bovine serum albumen/0.03% triton X-100/10 mM glycine). Brains were incubated overnight with primary antibodies in block buffer at 4°C. They were then washed for several hours in block buffer, incubated for one to two hours in secondary antibodies conjugated to rhodamine red-X or Cy5 (Jackson ImmunoResearch, West Grove, PA, USA), and washed several more times. Brains were equilibrated overnight in 85% glycerol/50 mM Tris pH 8 before mounting for microscopy. Primary antibodies used were: rabbit anti-GFP (Abcam, Cambridge, MA, USA), rabbit anti-Scrib [52], and mouse anti-CSP, kindly provided by Seymour Benzer.

### Analysis of fluorescence in larval brains

Larval brains were dissected and fixed as above, and either washed and transferred directly to glycerol, or processed for immunofluorescence. Whole brains were mounted in 85% glycerol/50 mM Tris pH8 under a coverslip and imaged on a BioRad (Hercules, CA, USA) Radiance 2100 confocal microscope. Images were processed using ImageJ [53].

### Generation of Wee-P insertions

Wee-P GFP transposon insertions were generated essentially as described [22]. GFP-positive embryos were manually picked using a dissecting microscope equipped for fluorescence (Olympus, Center Valley, PA, USA). GFP-positive fly lines were then screened for localized fluorescence either in the embryonic central nervous system or third instar larval brain. Wee-P insertion sites in lines of interest were identified by inverse PCR.

### Live imaging of the peripheral nervous system

Larvae with EB1-GFP or mCD8-GFP expressed in the peripheral nervous system were generated by crossing homozygous GFP transgene-containing lines with elav-Gal4 flies. Early second instar larvae were picked and placed on a slide with halocarbon 27 oil (Sigma, St Louis, MO, USA), then covered with a coverslip. Images were only collected for 10 minutes after mounting to ensure larvae were healthy. Images were collected at 166 lines per second, every 3.6 seconds, zoom 4 on a BioRad Radiance 2100 confocal microscope. Movies were compiled in ImageJ and Quicktime.

## Competing interests

The author(s) declare that they have no competing interests.

## Authors' contributions

MMR performed analysis of markers in larvae presented in the paper, and generated reagents needed unless specified below. DS generated EB1-GFP flies under the supervision of TU. PC designed the Wee-P screen and isolated some of the insertions shown. ALH oversaw the Wee-P screen performed in the Doe lab. CQD supervised and was involved in planning experiments. All authors read, made suggestions, and approved the final manuscript.

## Supplementary Material

Additional file 1 Determination of microtubule polarity in axons and dendrites using EB1-GFP dynamics. Flies containing an elav-Gal4 transgene were crossed to flies with a UAS-EB1-GFP transgene. Progeny were aged to early L2 and whole larvae were mounted for imaging. Single confocal images were acquired every 3.6 seconds. The movie can be viewed with Quicktime. Click here for file
